# American Academy of Dental Science

**Published:** 1873-01

**Authors:** 


*American Academy of Dental Science.*—The fifth Annual meeting of the American Academy of Dental Science was held in Union Hall, 300 Washington Street, Boston. The meeting was called to order by the President, Daniel Harwood, M. D., who made a few congratulatory remarks upon the attendance of members and concerning the progress of the Academy. A number of gentlemen were admitted to membership, and the Treasurer, Dr. E. G. Tucker, read a report, showing the healthy state of the finances. Officers for the ensuing year were elected as follows: President, Daniel Harwood, M. D.; Vice President,

Joshua Tucker, M. D.; Corresponding Secretary, E. N. Harris, D. D. S.; Recording Secretary, L. D. Shepard, D. D. S.; Treasurer, G. T. Moffatt, M. D.; Librarian, John Clough, M. D.; Censors, Drs. E. G. Tucker, J. L. Williams, and W. W. Codman. Prof. P. H. Austen, M. D., D. D. S., of the Baltimore College of Dental Surgery, who was to have delivered the annual address, sent a letter instead, in which he stated that the state of his health was such that it would be impossible for him to attend. He thus continued: "Permit me, before closing this letter of apology, to express my pleasure at the dignified position and bearing of your society. Also, let me accord my warmest approval of the educational movement in which some of you are engaged. In my unfinished address I have sought to show that the term liberal, as applied to any profession, should not be allowed to lose its classical meaning—that all professional schools should be so planned that their diplomas would necessarily imply familiarity with these liberal sciences which it is too much the custom of this practical age to regard as useless incumbrances of a gentleman's education. By bringing dental teaching within its university curriculum, Harvard designs, I take it, emphatically to indicate this specialty of medicine as one of the important practical applications of the power of educated mind. When a man is master of the arts of study and thinking, he is prepared, as he can in no other way be, to master the technical difficulties of his future profession. Hence, for one, I shall heartily rejoice when there exists neither medical nor dental schools, except in such connection with our literary colleges that a diploma from one implies graduation in the other. In no other way do I think we can stem the inundation of titled ignorance, which seems to be fast sweeping from the professions all trace of what once made them liberal."

Able essays were read as follows: By Dr. Jacob L. Williams, of Boston, on "The Influences of the Nervous System on the Health of the Teeth and Mouth." By Dr. Frederick N. Seabury, of Providence, R. I., on "Dentistry for the Poor, *vs.* Poor Dentistry." By Dr. George T. Moffatt, of Boston, on "An Improved Method of Treating Fractured Teeth." By Dr. Albion P. Stevens, of Portsmouth, N. H., on "Culture Demanded by a

Professional Life." By Dr. Charles E. Francis, of New York, on "The Rubber Dam." Interesting remarks were then made by various members of the Academy, and at 4 P. M. the annual dinner was partaken of at the Parker House.

